# Supplementary figures and images for: Establishment of the experimental procedure for prediction of conjugation capacity in mutant UGT1A1
Source: PLoS One. 2019 Nov 15;14(11):e0225244. doi: 10.1371/journal.pone.0225244 (PMC6857941; doi:10.1371/journal.pone.0225244)

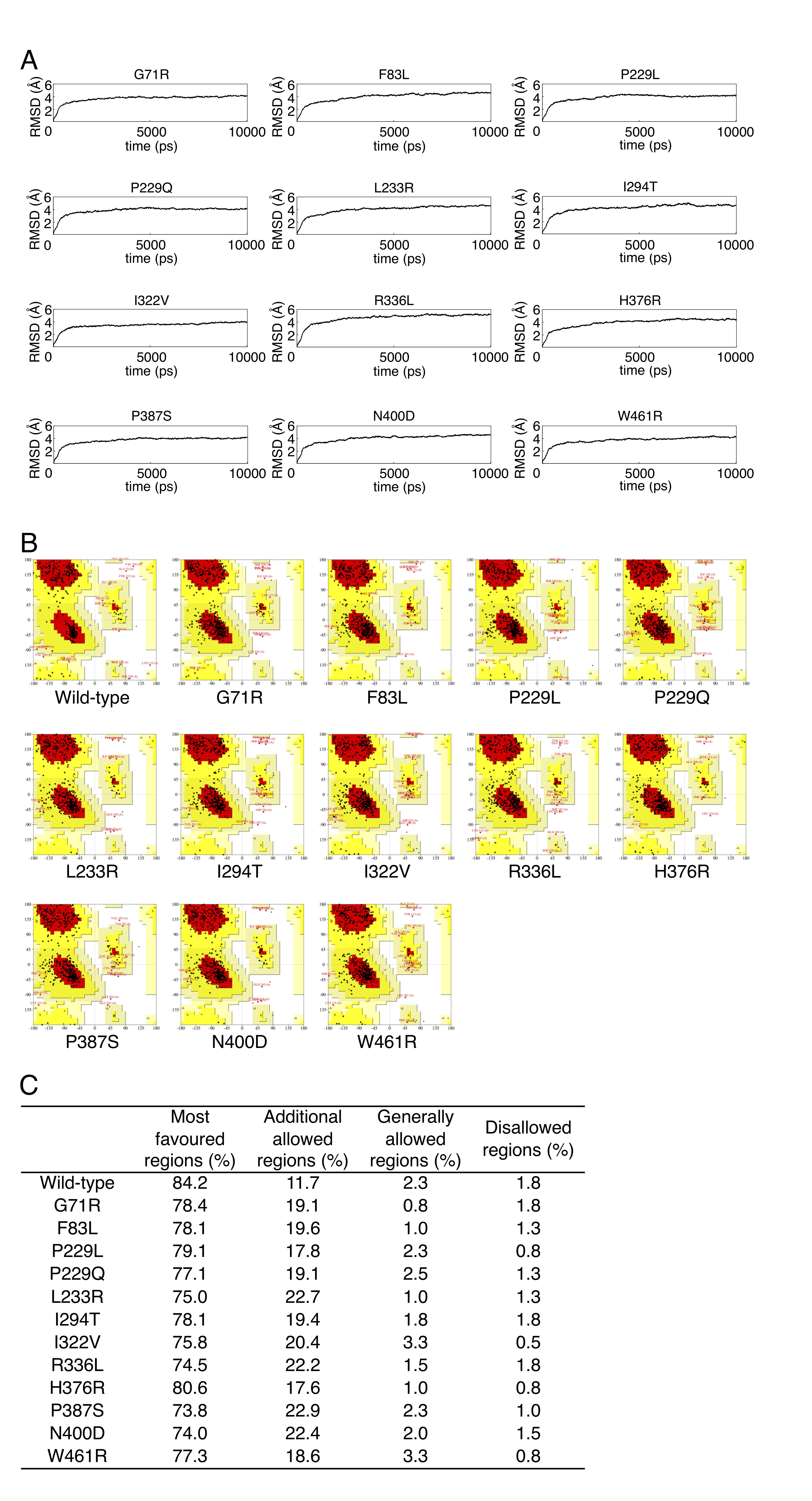

Supplement: S1 Fig — (A) The RMSD curves of the 10,000-ps trajectories for the backbone atoms of the UGT1A1 mutants were calculated with respect to the initial structures as a function of time. (B) Ramachandran plots for UGT1A1s. (C) Plot statistics for each UGT1A1. (TIFF) [file pone.0225244.s001.tiff]

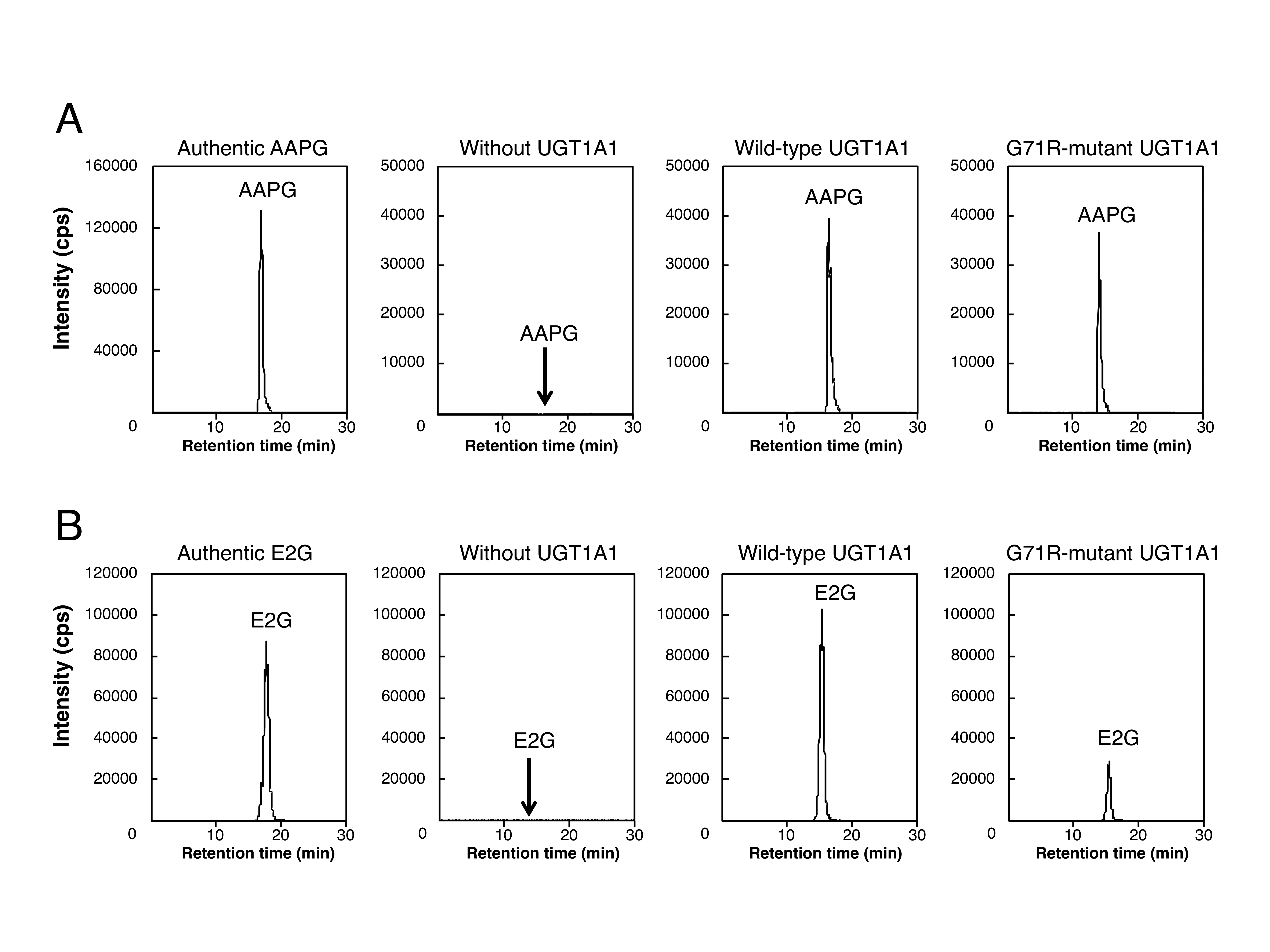

Supplement: S3 Fig — Representative chromatograms of (A) AAPG and (B) E2G in the reaction mixtures include authentic standard AAPG and E2G, reaction mixtures without UGT1A1, and mixtures with wild-type UGT1A1 or G71R-mutant UGT1A1. AAPG and E2G were detected in the reaction mixtures with wild-type UGT1A1 and G71R-mutant UGT1A1, whereas glucuronides were absent in the reaction mixtures without UGT1A1. (TIFF) [file pone.0225244.s003.tiff]

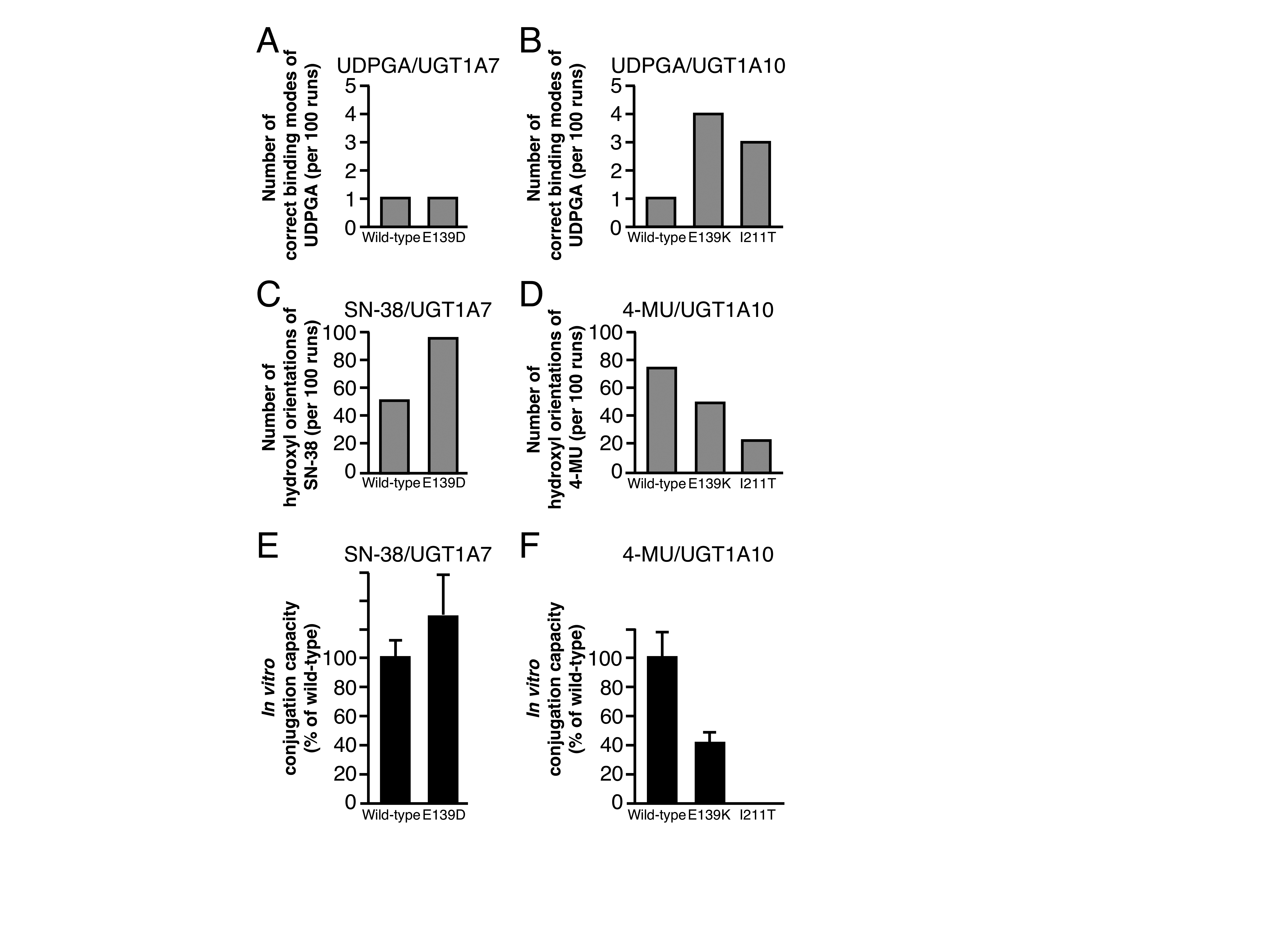

Supplement: S4 Fig — (A and B) The number of correct binding modes of UDPGA per 100 separate docking runs. (C and D) The number of correct binding modes (hydroxyl orientations) of substrate per 100 separate docking runs. (E and F) Reported in vitro conjugation capacity. A correlation was shown between the hydroxyl orientation of the substrate and the in vitro conjugation capacity. (TIFF) [file pone.0225244.s004.tiff]
