# Supplementary material for: Establishment of the experimental procedure for prediction of conjugation capacity in mutant UGT1A1
Source: PLoS One. 2019 Nov 15;14(11):e0225244. doi: 10.1371/journal.pone.0225244 (PMC6857941; doi:10.1371/journal.pone.0225244)
Supplement: S1 Table — (DOCX) [file pone.0225244.s005.docx]

| Atoms in hydrogen bonds  (VvGT - UDP) | A333/C - N21 | A333/N - O23 | E358/OE1 - O4 | E358/OE2 - OE6 | N354/N - O11 | S355/N - O12 | T280/OG1 - O17 |
| --- | --- | --- | --- | --- | --- | --- | --- |
| Hydrogen bond distance in the crystal structure (PDB ID: 2C1X) (Å) | 3.831 | 2.978 | 3.674 | 3.188 | 3.752 | 4.131 | 2.705 |
|  |  |  |  |  |  |  |  |
| Hydrogen bond distance in AutoDock results  (mean ± SD, n = 80) (Å) | 4.012 ±  0.551 | 3.162 ±  0.546 | 4.404 ±  0.402 | 3.414 ±  0.751 | 4.572 ±  1.424 | 4.680 ±  0.998 | 4.162 ±  1.342 |
|  |  |  |  |  |  |  |  |
| Difference between the crystal structure and the AutoDock results (Å) | 0.181 | 0.184 | 0.752 | 0.226 | 0.820 | 0.549 | 1.457 |
|  |  | | | | | | |
| Average difference of the hydrogen bond distance between the crystal structure and AutoDock result (mean ± SD) (Å) | 0.596 ± 0.465 | | | | | | |
