# Supplementary material for: Establishment of the experimental procedure for prediction of conjugation capacity in mutant UGT1A1
Source: PLoS One. 2019 Nov 15;14(11):e0225244. doi: 10.1371/journal.pone.0225244 (PMC6857941; doi:10.1371/journal.pone.0225244)
Supplement: S2 Table — (DOCX) [file pone.0225244.s006.docx]

| AAP | Wild-type | G71R | F83L | I322V | R336L | H376R | P387S |
| --- | --- | --- | --- | --- | --- | --- | --- |
| Number of correct binding modes of  UDPGA (per 100 runs) | 4 | 5 | 15 | 26 | 0 | 16 | 9 |
| Number of hydroxyl orientations of  AAP (per 100 runs) | 80 | 94 | 0 | 56 | N/A | 2 | 0 |
| E2 | Wild-type | G71R | F83L | I322V | R336L | H376R | P387S |
| Number of correct binding modes of  UDPGA (per 100 runs) | 4 | 5 | 15 | 26 | 0 | 16 | 9 |
| Number of hydroxyl orientations of  E2 (per 100 runs) | 70 | 16 | 17 | 70 | N/A | 5 | 3 |
| Bilirubin | Wild-type | G71R | F83L | P229Q | I294T | N400D | W461R |
| Number of correct binding modes of  UDPGA (per 100 runs) | 4 | 5 | 15 | 12 | 11 | 8 | 29 |
| Number of hydroxyl orientations of  bilirubin (per 100 runs) | 53 | 19 | 6 | 39 | 32 | 36 | 9 |
| SN-38 | Wild-type | G71R | P229L | P229Q | L233R |  |  |
| Number of correct binding modes of  UDPGA (per 100 runs) | 4 | 5 | 12 | 12 | 9 |  |  |
| Number of hydroxyl orientations of  SN-38 (per 100 runs) | 45 | 16 | 2 | 14 | 7 |  |  |
