# Supplementary material for: Establishment of the experimental procedure for prediction of conjugation capacity in mutant UGT1A1
Source: PLoS One. 2019 Nov 15;14(11):e0225244. doi: 10.1371/journal.pone.0225244 (PMC6857941; doi:10.1371/journal.pone.0225244)
Supplement: S3 Table — (DOCX) [file pone.0225244.s007.docx]

| Term | Description |
| --- | --- |
| *P_C_* | Predicted conjugation capacity |
| *S_DH_* | Number of hydroxyl orientations of the substrate |
| *S_DT_* | Total number of substrate docking |
| $\sigma$ | Substrate-specific constant |
| $\kappa$ | Constant that represents the TA repeat polymorphism in the promoter region |
| $\gamma_{l}$ | Gain of the sigmoid curve |
| $\mu_{l}$ | Midpoint of the sigmoid curve |
| $\varepsilon$ | Constant that represents the *in vivo* environment of enzyme reactions |
