# Supplementary material for: Establishment of the experimental procedure for prediction of conjugation capacity in mutant UGT1A1
Source: PLoS One. 2019 Nov 15;14(11):e0225244. doi: 10.1371/journal.pone.0225244 (PMC6857941; doi:10.1371/journal.pone.0225244)
Supplement: S1 Text — (DOCX) [file pone.0225244.s008.docx]

**S1 text. Analysis of the conformational difference between the docking model and the crystal structure.**

To evaluate the reproducibility of AutoDock [1] for the binding mode of the reported crystal structure, we used the docking analysis of the grape flavonoid 3-*O*-glucosyltransferase (VvGT) with UDP and compared its binding mode to that of the crystal structure. The 3D structure of the VvGT-UDP complex was obtained from the Protein Data Bank (PDB ID: 2C1X). After we added hydrogen atoms to the model structure via PyMOL software [2], 100 molecular docking runs of VvGT with UDP were performed with the AutoDock4 program. The docking site was defined by using the AutoGrid program according to the crystal structure (PDB ID: 2C1X). For each atom pair of VvGT and UDP, which formed a hydrogen bond in the crystal structure, the distance between the pair was analyzed and compared with that of the crystal structure. The correct binding mode of the VvGT-UDP complex was obtained in 80 of 100 docking runs. As S1 Table shows, the average difference in the hydrogen bond distances was 0.596 ± 0.465 Å longer in the docking results than that in the crystal structure (PDB ID: 2C1X).

**References**

1. Morris GM, Huey R, Lindstrom W, Sanner MF, Belew RK, Goodsell DS, et al. AutoDock4 and AutoDockTools4: automated docking with selective receptor flexibility. Journal of computational chemistry. 2009;30(16):2785-2791. Epub 2009/04/29. doi: 10.1002/jcc.21256. PMID: 19399780

2. Schrödinger LLC. The PyMOL Molecular Graphics System. 2.0 ed, 2017.
