# Supplementary material for: Establishment of the experimental procedure for prediction of conjugation capacity in mutant UGT1A1
Source: PLoS One. 2019 Nov 15;14(11):e0225244. doi: 10.1371/journal.pone.0225244 (PMC6857941; doi:10.1371/journal.pone.0225244)
Supplement: S2 Text — (DOCX) [file pone.0225244.s009.docx]

**S2 text. Correlation between docking simulation results and *in vitro* conjugation capacity of UGT1A7 and UGT1A10.**

We also analyzed SN-38 glucuronidation of UGT1A7 E139D and 4-MU (4-methylumbelliferone) glucuronidation of UGT1A10 E139K and I211T. The 3D structures of UGT1A7 and UGT1A10 were obtained from ModBase (Model IDs: UGT1A7,c5cd0160a7f889cbe74591844e9f2756; UGT1A10, 82ef85d3e55a142934a66e6a52a5a49), and 3D structures of substrates were obtained from ChemIDPlus (registry numbers: SN-38, 86639-52-3;4-MU, 90-33-5). Molecular simulation analyses were performed as described in the Materials and Methods section. As was the case with UGT1A1, the hydroxyl orientation of the substrate correlated with the reported *in vitro* conjugating capacity [1-3] (S4 Fig).

**References**

1. Dellinger RW, Fang JL, Chen G, Weinberg R, Lazarus P. Importance of UDP-glucuronosyltransferase 1A10 (UGT1A10) in the detoxification of polycyclic aromatic hydrocarbons: decreased glucuronidative activity of the UGT1A10^139Lys^ isoform. Drug metabolism and disposition: the biological fate of chemicals. 2006;34(6):943-949. Epub 2006/03/03. doi: 10.1124/dmd.105.009100. PMID: 16510539

2. Martineau I, Tchernof A, Belanger A. Amino acid residue ILE211 is essential for the enzymatic activity of human UDP-glucuronosyltransferase 1A10 (UGT1A10). Drug metabolism and disposition: the biological fate of chemicals. 2004;32(4):455-459. Epub 2004/03/25. doi: 10.1124/dmd.32.4.455. PMID: 15039300

3. Villeneuve L, Girard H, Fortier LC, Gagne JF, Guillemette C. Novel functional polymorphisms in the UGT1A7 and UGT1A9 glucuronidating enzymes in Caucasian and African-American subjects and their impact on the metabolism of 7-ethyl-10-hydroxycamptothecin and flavopiridol anticancer drugs. The journal of pharmacology and experimental therapeutics. 2003;307(1):117-128. Epub 2003/08/29. doi: 10.1124/jpet.103.054072. PMID: 12944498
