# Supplementary material for: Establishment of the experimental procedure for prediction of conjugation capacity in mutant UGT1A1
Source: PLoS One. 2019 Nov 15;14(11):e0225244. doi: 10.1371/journal.pone.0225244 (PMC6857941; doi:10.1371/journal.pone.0225244)
Supplement: Attachment — Submitted filename: reviewers_comments-and-answer.docx [file pone.0225244.s010.docx]

Response to reviewrs

We revised the manuscript based on the reviewers’ comments. Our english was corrected by a native professional editor. Our answers to the reviewers’ comments:

**Reviewer #1:**

**Major issues:**

**1. What was the normalizing control for transfection efficiency?**

Our answer:

We revised the sentence in line 176:

"...expression vectors, together with a luciferase reporter vector (pGL3 vector) as an indicator of transfection efficiency, ..."

**2. How were the glucuronide peaks confirmed for all substrates?**

Our answer:

The glucuronide peaks were confirmed because their retention times were the same as those of commercially available authentic standards.

In answer to your comment, we added the following sentences after line 205:

" The glucuronide peaks were detected at *m/z* 326.1/150.0 [precursor ion (Q1)/product ion (Q3)] for AAPG and *m/z* 447.1/271.1 (Q1/Q3) for E2G. These glucuronide peaks were …."

**3. The authors should show representative LC-MS/MS runs for all assays.**

Our answer:

We added S3 Fig, which shows representative LC-MS/MS chromatograms and cited this figure in line 207:

" S3 Fig shows representative LC-MS/MS chromatograms."

**4. What was the sensitivity of all of their assays? Minimal level of detection? Variability? How many times were each experiment run? Etc.**

Our answer:

We added the following information in the Materials and Methods section:

Line 205, "With regard to the sensitivity of all assays, the minimal level of detection was 3 ng/assay. Experiments were repeated five times; the coefficient of variation was less than 12%."

In addition, we revised the description of the LC-MS/MS procedures (lines 198 - 206) as follows:

"An API-3000TM LC-MS/MS system (Applied Biosystems/MDS SCIEX, Toronto, Canada) was ……… repeated five times; the coefficient of variation was less than 12%."

**5. P. 10. Line 165. What SDM method was used?**

Our answer:

Thank you very much for your comment, but we have already described our method and cited our previous paper to refer to that method (line 166 – 167, ref No. 35).

**6. P. 11. Line 177. What is the ‘UGT reaction mix’? This reviewer is not confident about the use of this procedure and it providing accurate results.**

Our answer:

We added the following information to line 181:

"…the UGT Reaction Mix containing UDPGA (Becton, Dickinson and Company, Franklin Lakes, NJ, USA)"

**7. Lines 185-187. Why were PNPG and EE2 added to the reaction mix?**

Our answer:

We changed the sentence in line 193 in the Materials and Methods section:

"After the reaction termination, 5 μL of 1mM *p*-nitrophenyl β-d-glucuronide (PNPG) ….. as an internal standard."

**8. Too many supplemental tables and figures.**

Our answer:

We merged S1 and S2 Figs to new S1 Fig.

**9. Fig. S1 is not well-explained.**

Our answer:

We added the following information to the S1 Fig legend in line 554:

" S1 Fig. Validation of the 3D structures of wild-type UGT1A1 and ….. for UGT1A1s. (C) Plot statistics for each UGT1A1."

**10. Fig. S3 is not sufficiently clear.**

Our answer:

We improved the resolution of images in S3 Fig, which is now S2 Fig.

**11. Table S1 is not properly formatted.**

Our answer:

We revised S1 Table.

**P. 7. Line 100. Is solvate a word?**

Our answer:

We changed the sentence in line 103-105:

" The transferable intermolecular ….. of UGT1A1 with water."

**Line 120. Add ‘The’ at the beginning of the sentence.**

**Do not put figure legends within the text of the manuscript – makes it difficult to read.**

Our answer:

We placed the figure legends according to the journal’s Submission Guidelines for authors: “Figure captions must be inserted in the text of the manuscript, immediately following the paragraph in which the figure is first cited (read order).”

**P. 10. Line 164. Delete the use of the term ‘entry clone’. Just say that the vector was used to generate a clone….**

Our answer:

We modified the sentence in line 169:

"Human UGT1A1 cDNA was obtained from a human liver cDNA library by PCR amplification and was inserted into a pENTR/D-TOPO vector. "

**Line 169. Poor sentence.**

Our answer:

We revised the sentence in line 173-174:

"Each wild-type and mutant UGT1A1 sequence was inserted into the expression vector pcDNA-DEST40 Gateway by recombination."

**Table 1. The UDPGA rows are repetitive and unnecessary. The amino acid changes headers are also repetitive and unnecessary.**

Our answer:

According to your suggestion, we revised Table 1, which is now S2 Table.

**Table 3. Should be a supplemental table as it does not provide data.**

Our answer:

Table 3 is now S3 Table.

**Table 4. The same should be followed for Table 4 as described above for Table 1. For Table 4, why were no data provided for N400D and W461R – this should at least be explained in a footnote within the table.**

Our answer:

We added the sentence in line 230-232:

"The docking analyses were ….. capacities were reported."

**P. 23. Line 353. You do not need this sentence.**

Our answer:

We removed the following sentence.

**Line 359. Poor sentence structure.**

Our answer:

We revised the sentence in line 366-368:

"To derive our mathematical model, we both ……. of the mutant UGT1A1s."

**Lines 366, 378, 546, 548, 550. Should read, ‘UGT1A enzymes…’.**

Our answer:

According to your suggestion, we modified the sentence in line 373:

"UGT1A enzymes ..."

We also changed line 385 to read " all UGT1A1 mutants based on Figure 6" and also line 555 to read "UGT1A1 mutants."

**Lines 381-83. Don’t need this sentence.**

Our answer:

We removed the following sentence.

**Lots of English to correct, including the following:**

**P. 4. Line 51. Fix ‘UGT1A1s’. Change ‘especially’ to ‘including’.**

**Line 53. reported ‘on a’ binding model.**

**Lines 71. ‘sequence’ to ‘sequencing’.**

**Lines 77-79. Not great English.**

**Line 79. The word ‘concretely’ is not meaningful here.**

**Line 81. Change to ‘We subsequently developed…’.**

**P. 6. Line 98. Delete ‘by’.**

**P. 11. Line 171. ‘…used for UGT1A1 activity and luciferase assays’.**

**P. 13. Line 210. Use ‘We removed one of the UGT1A1 mutants…’**

**Line 218. Add ‘and’ L233R.**

**P. 14. Line 225. Finally, ‘a’…**

**Line 238. You are comparing the conjugation capacity of UGT1A1 against AAP and E2 with…**

**Line 112. Delete ‘We can see that’, and change ‘is’ to ‘was’.**

**P. 8. Line 117. Change ‘ following’ to ‘docking’**

**Line 364-65. Should be, ‘In our docking analysis using the AutoDock…’.**

**Line 368. Should read’ ‘including the number…’.**

Our answer:

We edited according to the reviewer’s comments.

**Reviewer #2:**

**1. It would be helpful to define "molecular simulation” because it was challenging to determine whether it was synonymous with “in silico” throughout the paper.**

Our answer:

We used the term "molecular simulation" for structural optimization and/or docking analysis. We used the term "*in silico*" for analyses including "molecular simulation" and the mathematical model. Please see lines 74, 248, 360-361, 362 and 379.

For a more accurate description, we changed "molecular simulation" in line 250 to read "docking simulation."

In addition, we used the term "*in silico*" as an antonym for "*in vitro*" in lines 85, 223, 238, 336, and 368.

**2. Line 54, please add “amino acids” before listing them so the reader knows what the list is.**

Our answer:

We added the words “amino acids” in lines 56 and 135:

"... amino acids S38, H173, G308, L355, S375, H376, and G377, ….."

**3. Line 99, please define TIP3P.**

Our answer:

We corrected the sentence in line 103-105:

" The transferable intermolecular potential ….. structural optimization of UGT1A1 with water."

**4. Line 104, please define MM.**

Our answer:

We defined MM in the sentence in line 108-111:

"We performed energy minimization by means of the molecular mechanics (MM) function of the MOE software..."

**5. I can’t see the Figure 1 images. Please enhance and possibly enlarge.**

Our answer:

We fixed Fig 1:

(1) We enlarged the images.

(2) We showed typical docking results of wild-type UGT1A1 and each substrate.

(3) We corrected the figure legend in line 154.

We also corrected the sentence in line 151:

"Fig 1 The complexes of UGT1A1 with coenzyme and substrates."

**6. The location of the figure legends makes the text a little tougher to follow. It would be easier as a reviewer to have the figure legends at the end of the document with the figures. The first section of results is broken up by one figure legend and two tables, making it more work for the reader to figure out what the block of text is associated with.**

Our answer:

We placed the figure legends according to the journal’s Submission Guidelines for authors: “Figure captions must be inserted in the text of the manuscript, immediately following the paragraph in which the figure is first cited (read order).”

**7. There are several inconsistencies relating to the figures. For instance, when referenced within the text in parentheses, most times it appears as (fig X) and at least once, it appears as (fig. X). There are also spaces between figure numbers and panel letter in places and not in others. In the figure legends, some are listed as Panels “A,B” and other times as (A), (B).**

Our answer:

We changed the following citations:

Line 279-280: fixed the sentence to read "Fig 2B and 2C"

Line 316: fixed the sentence to read "Fig 3A and 3B"

Line 332: fixed the sentence to read "Figs 4, 5A, and 5B"

**8. It would be helpful for Table 1 and Figure 2 to be near each other so that they can be viewed together.**

**Figures 2 and 3 are showing the same data for acetaminophen and 17-B-estradiol in separate graphs and then in a single graph. The pattern is not repeated for the bilirubin and SN-38. I recommend the authors be consistent and either do both graphs for bilirubin and SN-38 as well or just show figure 3 for the acetaminophen and 17-B-estradiol.**

Our answer:

Please see line 332 and 339. And we added Fig 4, which shows docking results and *in vitro* conjugation capacities of wild-type UGT1A1 and UGT1A1 mutants for bilirubin and SN-38.

**9. It would be helpful for the authors to add a small figure legend within the graphic that says what the grey and black bars are for figures 3 & 4. Also, a small figure legend within figure 5 listing each of the identities of the symbols and the r value and slope.**

Our answer:

We added a small key to these figures to explain the symbols.

We also added the slope, *R* value, and *P* value to the figure 6.

The original Figs 3, 4, and 5 are now renumbered as Figs 3, 5, and 6.

**10. I would like to see a citation for the sentence in lines 346-347. This may just be an oddly worded sentence since the text of the article is about mutations and polymorphisms that alter amino acids in the protein sequences; however, it should be either cited or altered.**

Our answer:

We corrected the sentence in line 353-355:

"Recently, novel mutations causing amino acid substitutions have been found by genome analysis with high-throughput sequence technology [43]."

**11. It would be more impactful if the authors stated in the results section which correlations were statistically significant. It may be that all correlations mentioned in the results were statistically significant, but it still makes it more work for the reader to figure that out.**

Our answer:

We added "significant" before the word "correlation" in lines 251, 316, 332, and 336.
