# Supplementary material for: Establishment of the experimental procedure for prediction of conjugation capacity in mutant UGT1A1
Source: PLoS One. 2019 Nov 15;14(11):e0225244. doi: 10.1371/journal.pone.0225244 (PMC6857941; doi:10.1371/journal.pone.0225244)
Supplement: Attachment — Submitted filename: Response to Reviewers.docx [file pone.0225244.s011.docx]

Response to Reviewers’

We revised the manuscript under the editors’ comments.

Our answers to the comments are as follows:

**Editor's Comments:**

**1. Line 59: Please clarify the term 'newborn diseases'. Do you mean diseases in newborns? Or new diseases?**

Our answer:

We revised the sentence in line 59:

"...found in hereditary diseases, ..."

**2. Line 66: Please clarify the UGT1A1*28 allele is the promoter.**

Our answer:

In answer to your comment, we clarified the UGT1A1*28 allele is the promoter in line 66:

" …in the TA repeat from 6 to 7 of the promoter region."

**3. Lines 72-74: Can you be more specific what is being conjugated for better clarify.**

Our answer:

We added bilirubin and SN-38 for clarify and rewrote correctly in line 73:

"…these new mutant UGT1A1s for any substrate, such as SN-38 are usually unknown."

**4. Lines 75-76: Can you be more specific what is being conjugated for better clarify.**

This sentence means that the molecular simulation analysis is a powerful tool for studying the biochemical process. So, we delete "of conjugation" in line 75, which existed in the end of the sentence before:

Line 75, "…. became a powerful tool for studying the biochemical process."

**5. Line 81: Please be more specific for what is being conjugated in this context.**

Our answer:

We rewrote for clarify in line 81 to 83:

"…we revised our previous molecular simulation analyses, which led to a new mathematical model for the conjugation process in order to predict the conjugation capacity for substrates such as bilirubin and SN-38."

**6. Line 85: Are there previous models for this? If so, please provide references.**

Our answer:

We deleted "new" in line 82: Before this study, there was no mathematical model for prediction of the conjugation capacity by UGT1A1. We used this "new" for an initial model.

And we rewrote to line 87:

" Then we demonstrate here that…"

**7. Line 163: Please define why this mutant was selected for Figure 1F. Is this just**

**a represented graph or a different reason for looking at this particular variant.**

Our answer:

We changed and added the sentences for the explanation of Fig1F in line 152 to 155:

"Figs 1A, 1B, 1C, and 1D show representative docking results of hydroxyl orientations and other orientations that cannot function in the conjugation reaction. Fig 1E shows the field of the conjugation process in wild-type but the field completely lacks in R336L mutant (Fig 1F)."

**8. Line 167: No paragraph indent needed.**

Our answer:

We edited according to the editor’s comments. Please see line 170.

**9. Line 310: Please rewrite to clarify: “S2 Table show the variables and constants**

**of the mathematical model for the docking results for each UGT1A1 variant.” or**

**similar so that it is more clear.**

Our answer:

We edited according to the editor’s comments and added the sentence to clarify in line 312 to 313:

"S2 Table shows the variables and constants of the mathematical model for the docking results for each UGT1A1 variant."

**10. Lines 334-335: This should be clarified to "validated for each tested**

**metabolite." And the paragraph indentation can be eliminated for the next**

**sentence to create one paragraph.**

Our answer:

We edited according to the editor’s comments, created one paragraph, and added the sentence to clarify in line 336 to 340:

"These results indicate that our method for the prediction of conjugation capacity is valid in the tested cases of bilirubin and SN-38. Figure 6 indicates that our experimental procedure is effective for predicting the conjugation capacity, because all *in silico* results and *in vitro* results were significantly correlated."

**11. Line 339-340: Figure 4 legend does not appear to have text outside of the title**

**(assuming similar to text in figure 2 legend).**

Our answer:

We added the legend according to the editor’s comments in line 344 to 347:

"(A) The number of correct binding modes of UDPGA per 100 separate docking runs. (B and C) The number of hydroxyl orientations of substrate per 100 separate docking runs. S2 Table provides raw data for docking results for panels A, B, C, and D. (E and F) In vitro conjugation capacity."

**12. Line 348: There does not appear be text for Figure 6 outside of a title.**

Our answer:

We added the figure legend in line 356:

" Regression equation (solid line), Pearson's correlation coefficient (R), and P value are shown."

**13. Line 360: Should be procedures, not procedure**

Our answer:

We edited according to the editor’s comment in line 368.

**14. Line 368: Should be clarified to say several categorized mutant UGT1A1s.**

Our answer:

We clarified the sentence in line 376:

" … of G71R, F83L. I322V, R336L, H376R, and P387S mutant of UGT1A1s."

**15. Line 385: Are these the only known UGT1A1 variants, or just the most studied?**

**Please clarify.**

Our answer:

We revised the sentence to clarify for the variants in line 392 to 395:

"In this research, our procedure demonstrates the potential to predict the capacity of all UGT1A1 mutants based on Figure 6, which shows the data of the most studied mutants *in vitro*, and it also suggests that the same strategy may be available for other metabolic enzymes."

**16. Table S1: Please add in guidelines to this table so that the values for the**

**hydrogen bars and their mean A distance are easier to read/categorize for each**

**category.**

Our answer:

We added the guidelines in S1 Table according to the editor’s comment.
